# Modeling ocean-induced rapid Earth rotation variations: an update

**DOI:** 10.1007/s00190-021-01555-z

**Published:** 2021-09-07

**Authors:** Alexander A. Harker, Michael Schindelegger, Rui M. Ponte, David A. Salstein

**Affiliations:** 1 Institute of Geodesy and Geoinformation, University of Bonn, Nussallee 15, 53115 Bonn, Germany; 2 Atmospheric and Environmental Research, Inc., Lexington, MA USA

**Keywords:** Earth rotation, Geophysical fluids, Excitation, Ocean bottom pressure

## Abstract

We revisit the problem of modeling the ocean’s contribution to rapid, non-tidal Earth rotation variations at periods of 2–120 days. Estimates of oceanic angular momentum (OAM, 2007–2011) are drawn from a suite of established circulation models and new numerical simulations, whose finest configuration is on a $^\circ$ grid. We show that the OAM product by the Earth System Modeling Group at GeoForschungsZentrum Potsdam has spurious short period variance in its equatorial motion terms, rendering the series a poor choice for describing oceanic signals in polar motion on time scales of less than $\sim$2 weeks. Accounting for OAM in rotation budgets from other models typically reduces the variance of atmosphere-corrected geodetic excitation by $\sim$54% for deconvolved polar motion and by $\sim$60% for length-of-day. Use of OAM from the $^\circ$ model does provide for an additional reduction in residual variance such that the combined oceanic–atmospheric effect explains as much as 84% of the polar motion excitation at periods < 120 days. Employing statistical analysis and bottom pressure changes from daily Gravity Recovery and Climate Experiment solutions, we highlight the tendency of ocean models run at a 1$^\circ$ grid spacing to misrepresent topographically constrained dynamics in some deep basins of the Southern Ocean, which has adverse effects on OAM estimates taken along the 90$^\circ$ meridian. Higher model resolution thus emerges as a sensible target for improving the oceanic component in broader efforts of Earth system modeling for geodetic purposes.

## Introduction

**Fig. 1:**
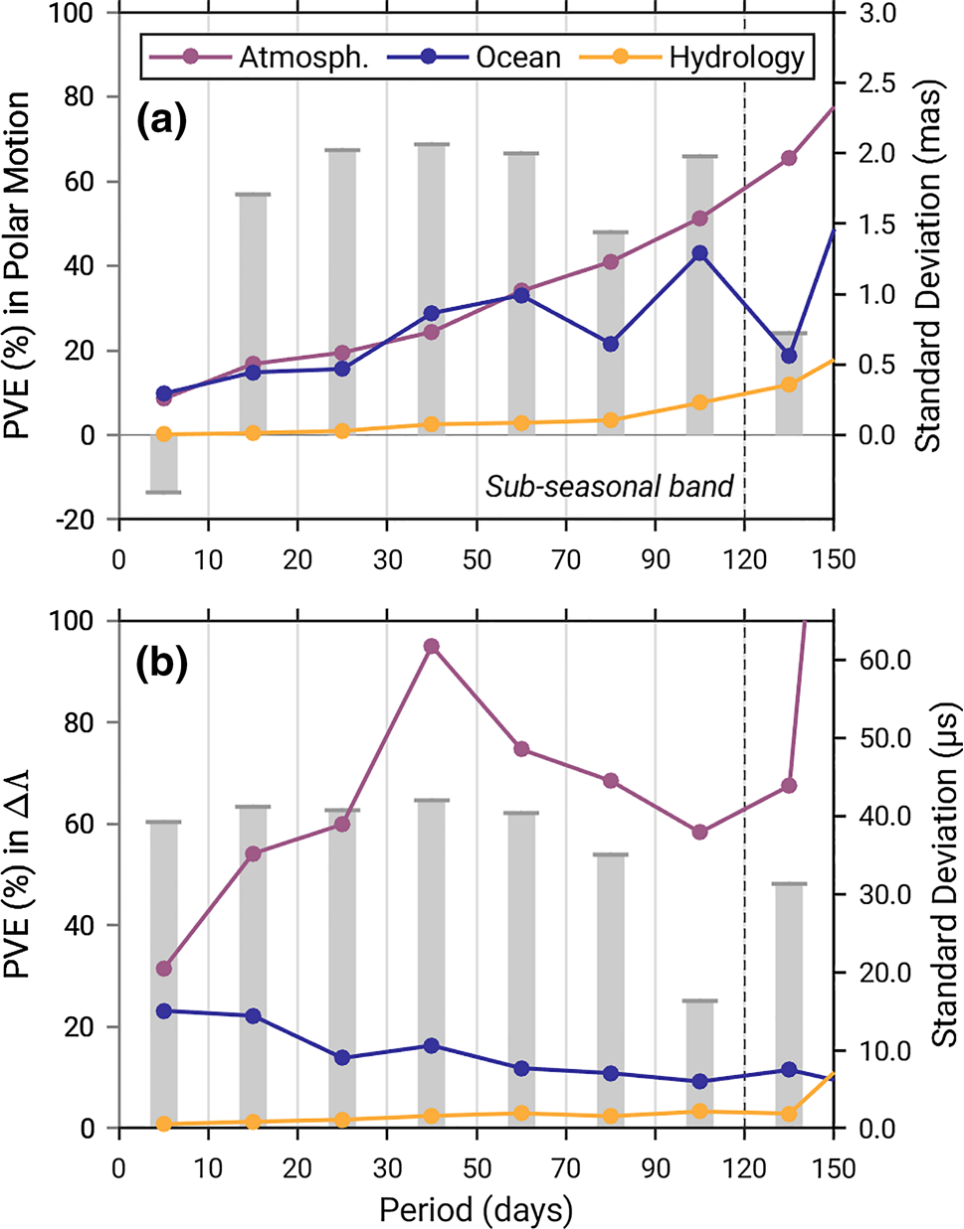
Overview of sub-seasonal non-tidal variability in daily-sampled **a** polar motion (mas) and **b** ${\Delta \Lambda }$ ($\upmu$s). Solid lines are standard deviations of atmospheric (purple color), oceanic (dark blue), and hydrological (yellow) contributions to the respective Earth rotation parameter, separated into eight disjoint intervals from $\left[ 2,10\right]$ days through $\left[ 120,150\right]$ days. Vertical bars, referred to by the left axes, show the percentage of variance explained (PVE) in complex-valued polar motion (not polar motion excitation) and ${\Delta \Lambda }$ by the ocean, after subtraction of atmospheric contributions. Angular momentum estimates are from Dobslaw and Dill (2018) and span the time period 2007–2011. Section 3.5 describes the Earth rotation data and relevant tidal corrections

The ocean stores and releases appreciable amounts of non-tidal angular momentum. Work around the turn of the millennium (Ponte et al 1998; Marcus et al 1998; Johnson et al 1999; Nastula and Ponte 1999; Chen et al 2000; Wünsch 2000) demonstrated that these kinematic fluctuations cause measurable changes in the rotation of the solid Earth on time scales from days to years. Subsequent analyses (e.g., Ponte and Ali 2002; Gross et al 2003, 2004; Zhou et al 2005; Bizouard and Seoane 2010) refined our quantitative understanding of these effects and their relative importance compared to rotational contributions (i.e., “excitations”) by other geophysical fluids. As a paramount example, Fig. 1 synthesizes variability in both polar motion and excess length-of-day (${\Delta \Lambda }$) associated with atmospheric, oceanic, and hydrological angular momentum (AAM, OAM, HAM), from periods *T* of 2 to 150 days. This “sub-seasonal” end of the spectrum is unique in that relevant excitations arise from either atmosphere or ocean, in an approximate ratio of 2:1 for polar motion at $T<$ 120 days (Gross et al 2003). As opposed to longer (e.g., annual) periods, requirements for a consistent treatment of gravitational attraction and mass balance effects at such time scales are weak (Quinn et al 2015), and the benefit of incorporating HAM estimates is small or unclear (Jin et al 2012; Quinn et al 2019). Thus, sub-seasonal periodicities constitute an almost ideal testing ground for closure in Earth’s rotational budget and the fidelity of atmosphere–ocean angular momentum estimates.

In this light, Fig. 1 also defines our problem. Modern fluid angular momentum series—here taken from Dobslaw and Dill (2018)—leave significant fractions of observed rapid polar motion and length-of-day changes unexplained. Specifically, with atmospheric effects removed, residual rotational variance accounted for by a numerical ocean model amounts to 60–65% at best, cf. Quinn et al (2019) for a similar, though more qualitative, assessment based on another OAM product. Although geodetic observations and AAM estimates have inherent uncertainties (e.g., Meyrath et al 2013; Schindelegger et al 2013; Ray et al 2017; Dill et al 2020), ocean models are a more likely source of error in such comparison. In particular, model-data syntheses that obey kinematic consistency (Wunsch and Heimbach 2013) must cope with serious gaps in the ocean observing system so that data adjustment intervals are in the order of weeks (Quinn et al 2019). Classical forward models can substitute some of this void and are integral to predictive frameworks (e.g., Dill et al 2019). However, the quality of forward OAM estimates varies with the adopted boundary conditions (e.g., forcing, bottom topography) and numerical choices pertaining to dissipative closures and discretization.

To make progress, it seems worth testing rotational excitation budgets with a wider set of OAM series, sampling different aspects of the ocean modeling parameters space. Our analysis emphasizes sub-seasonal periods, i.e., $T<$ 120 days, but insights into how to reduce structural errors in forward models may be similarly relevant to longer time scales and situations where a physically consistent fit to oceanographic data (e.g., bottom pressure estimates from satellite gravimetry) is desired. In what follows, we introduce the broad strokes of the excitation formalism (Sect. 2), describe our collection of ocean models (Sect. 3), present results (Sect. 4), and conclude with a summary and an outlook on further lines of research.

## Basic considerations

Conservation of a planet’s angular momentum provides a convenient framework to relate observed changes in Earth rotation to mass motions and redistributions in geophysical fluids (Munk and MacDonald 1960). In a rotating, geocentric terrestrial reference frame (TRF), having its *x* and *y* axes aligned with the Greenwich and 90$^\circ$ meridians, linearization of the governing equations in the equatorial direction gives (e.g., Brzeziński 1992)

1 $$\begin{aligned} {\hat{p}} + \mathrm {i} {\hat{\sigma }}^{-1}_c \dot{{\hat{p}}} = {\hat{\chi }} \end{aligned}$$

where ${\hat{p}} = {\hat{p}}(t) = x_p - \mathrm {i} y_p$ ($\mathrm {i} \equiv \sqrt{-1}$) is the reported position of the conventional reference pole in the TRF, the dot indicates derivative with respect to time *t*, and ${\hat{\sigma }}_c = 2\pi \left( 1 + \mathrm {i}/2Q \right) /T_c$ is the complex-valued Chandler frequency defined by period $T_c=433.0$ days and quality factor $Q=179$ (Wilson and Vicente 1990; Gross et al 2003). ${\hat{\chi }} = {\hat{\chi }}(t) = \chi _x + \mathrm {i}\chi _y$ represents the equatorial excitation function (sometimes called “effective angular momentum function”) of the respective geophysical fluid, computed using formulae given below. In practical analyses, one determines a geodetic excitation function from the deconvolution of pole path observations on the left side of Eq. (1) and budgets it against a linear combination of fluid excitations. Upon Fourier transformation, Eq. (1) becomes (Brzeziński 1992)

2 $$\begin{aligned} {\hat{p}}(\sigma ) = {\hat{\sigma }}_c \left( {\hat{\sigma }}_c - \sigma \right) ^{-1} {\hat{\chi }}(\sigma ) = {\hat{T}}(\sigma ){\hat{\chi }}(\sigma ) \end{aligned}$$

with $\sigma$ denoting frequency and ${\hat{T}}(\sigma )$ being a transfer function that convolves the excitation with the Chandler wobble response. This integration method underlies Fig. 1a, but its use for annual and lower frequencies is generally discouraged given the random-walk nature of the free wobble (Chao 1985). In the axial direction, we have (Rosen and Salstein 1983)

3 $$\begin{aligned} {\Delta \Lambda } = \chi _z \cdot 86400~s \end{aligned}$$

where ${\Delta \Lambda }$ is referred to as excess length-of-day, i.e., the incremental revolution time of the Earth with respect to an SI-day.

The dimensionless quantities $\left( {\hat{\chi }},\chi _z \right)$ encapsulate fluid angular momentum changes, arising from the redistribution of matter (mass term) and the movement of particles relative to the solid body rotation (motion term). As usual, let ${\hat{c}} = c_{xz} + \mathrm {i} c_{yz}$ and $c_{zz}$ be equatorial and axial perturbations of Earth’s inertia tensor and $\left( {\hat{h}},h_z \right)$ be relative angular momentum changes of the fluid layer. The excitation functions then take the form (e.g., Dobslaw and Dill 2019, and references therein)45where ${\Omega }$ is the mean sidereal rotation rate, *C* and *A* are polar and average equatorial moments of inertia of the entire Earth, and the numerical constants derive from rheological considerations. Implicit in this formulation are the (very accurate) assumptions of a rotationally symmetric Earth and complete core–mantle decoupling on sub-seasonal time scales; see Chen et al (2013) for possible refinements of the theory.

Superscript *v* in Eqs. (4)–(5) signifies the motion (velocity) term, which must be calculated from volume integrals over horizontal velocities weighted by density (e.g., Barnes et al 1983; Gross et al 2003, 2004). All matter terms (superscript *m*) used in this study are based on area integrals of mass loads, with atmospheric pressure ($p_a$) values over oceans replaced by their spatial average $\overline{p_a}$ according to the inverted barometer (IB) effect (Ponte 1999). The time series of $\overline{p_a}$, a static contribution to ocean bottom pressure $p_b$, is then taken to represent atmospheric not oceanic excitation. In most models, IB-corrected values of $p_b$ will have a nonzero time-dependent global mean, carrying both true mass changes and artificial $p_b$ variability associated with the Boussinesq approximation, cf. Ponte (1999). An ad hoc correction ensuring ocean mass conservation is to add a global uniform layer of the necessary time-varying thickness to the sea surface and adjust the mass element in the OAM integration (Gross et al 2004; Quinn et al 2019). Equivalently, one can reduce $p_b$ at latitude $\phi$ and longitude $\lambda$ to a dynamic residual $p_b'$ per time step

6 $$\begin{aligned} p_b'\left( \phi ,\lambda \right) = p_b\left( \phi ,\lambda \right) - A^{-1}\int _{A}^{}p_b\left( \phi ,\lambda \right) \cos \phi \mathrm {d}\phi \mathrm {d}\lambda \end{aligned}$$

(*A* is the surface area of the global ocean) and cast the net freshwater flux into the ocean due to precipitation, evaporation, and continental run-off in an additional, barystatic sea-level angular momentum function (SLAM, Dobslaw and Dill 2019; Dill et al 2019). As such, SLAM manifests an overall atmosphere–hydrosphere mass conservation constraint, which is essential for closing the annual ${\Delta \Lambda }$ budget (Yan and Chao 2012). With the net mass contributions to OAM accounted for by external estimates (see next section), all oceanic mass terms in our study reflect dynamic bottom pressure variability (Eq. 6).

Throughout the paper, we interchangeably use the terms “excitation function” and “angular momentum function” (Eqs. 4 and 5) and indicate the respective subsystem by the prefix (e.g., OAM function). A corresponding symbolic notation is $\chi _j^A$ for atmospheric (surface pressure and winds), $\chi _j^O$ for oceanic (bottom pressure and currents), and $\chi _j^G$ for geodetically observed excitation, where $j=\left( x,y,z\right)$. For convenience, we convert equatorial excitation functions from (rad) to (mas) and adopt units of ($\upmu$s) in considerations of the axial component (Eq. 3).

## Models and data sets

Our analysis spans a full 5-year period (2007–2011), which allows for robust inferences about rapid ERP variability and renders our own simulations (Sects. 3.3, 3.4) manageable. We examine OAM series and related excitation functions from four volume-conserving Boussinesq ocean models and one barotropic (2D, constant-density) model. Forcing fields across the simulations share a common origin in the European Centre for Medium-Range Weather Forecasts (ECMWF) archive, with ERA-Interim data (Dee et al 2011) adopted in three cases. All models include $\nabla p_a$ in the horizontal momentum equations, meaning that the simulated OAM changes are also impacted by dynamic signals associated with departures from the IB response. Periodic oscillations at diurnal and semi-diurnal frequencies were either removed from the barometric pressure fields prior to the simulations (ECCO Consortium et al 2020) or during post-processing of bottom pressure and horizontal velocities (Dobslaw and Dill 2018). We work with daily excitation time series, obtained from averaging 6-hourly (sometimes 3-hourly) mass and motion terms to a center time of 12 UTC.

### MPIOM

The Earth System Modeling Group at GeoForschungsZentrum Potsdam (ESMGFZ, Dobslaw and Dill 2018) provides geophysical excitation functions (AAM, OAM, HAM, SLAM) from atmosphere–hydrosphere models that are coupled through a global mass balance constraint. The oceanic component within this concerted approach is the Max-Planck-Institute for Meteorology Ocean Model (MPIOM Jungclaus et al 2013), discretized on a 1$^\circ$ tripolar grid and 40 vertical layers. The model is forced with operational ECMWF analysis fields, including instantaneous surface stresses calculated offline from horizontal winds at 10 m and boundary layer stability theory (Dobslaw and Dill 2019). For consistency, we use the MPIOM-based OAM functions in conjunction with atmospheric excitation series from operational ECMWF products, rather than ERA-Interim as for the models described below. The hydrology component in the ESMGFZ framework is supplied by the Land Surface Discharge Model (LSDM, Dill 2008), and SLAM estimates are derived from excess masses of LSDM and ECMWF at 24-hour intervals. The ESMGFZ set of angular momentum functions is well curated and has been widely used for studying various aspects of Earth rotation variability (Ray and Egbert 2012; Dill et al 2019; Ron et al 2019; Yu et al 2021).

### ECCOv4

ECCO (Estimating the Circulation and the Climate of the Ocean) Version 4 Release 4 (ECCOv4 for short) is a global ocean state estimate derived using adjoint-based nonlinear inverse modeling (Forget et al 2015; ECCO Consortium et al 2020). ECCO estimates are exact solutions to the Massachusetts Institute of Technology general circulation model (MITgcm, Marshall et al 1997), fitted iteratively to a large amount of oceanic in situ and satellite data. As such, it is the only “model” used here to be constrained to observations, including monthly bottom pressure anomalies from GRACE (Gravity Recovery and Climate Experiment, JPL RL05 mascons version 2, Watkins et al 2015). Along with the model’s initial conditions and uncertain internal parameters, the fitting procedure also corrects atmospheric forcing fields, with mean adjustments calculated over 14-day periods and interpolated to the model time step. The resultant forcing fields then drive a free forward integration of the MITgcm. The solution is defined on a curvilinear grid in which an Arctic cap transitions southward into a regular latitude–longitude grid (Lat-Lon-Cap or LLC grid). The configuration germane to ECCOv4 has a nominal $\sim$1$^\circ$ resolution about the equator and thus takes the name LLC90 (Forget et al 2015). We primarily use the solution’s OAM series without freshwater-induced surface loads, made available at the IERS SBO (International Earth Rotation and Reference Systems Service, Special Bureau for the Oceans) site (https://isdc.gfz-potsdam.de/ggfc-oceans/, accessed 28 December 2020). Note that Quinn et al (2019) assessed non-tidal OAM estimates from a previous ECCOv4 release that did not incorporate the effects of atmospheric pressure loading.

**Fig. 2:**
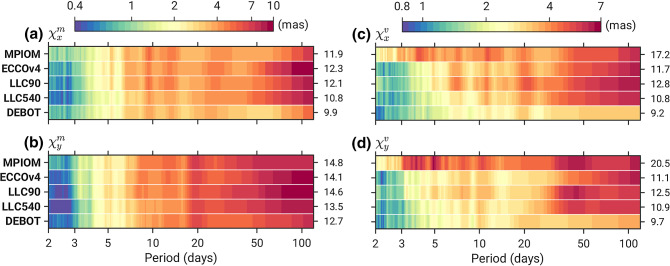
Amplitude spectra for equatorial **a**, **b** mass terms $\chi ^m_{x,y}$ and **c**, **d** motion terms $\chi ^v_{x,y}$ (in mas) from the five analyzed models, for periods 2–120 days. Estimates of power *P* were obtained from a 512-point Fast Fourier Transform using Welch (1967)’s method and converted to amplitudes $\sqrt{2 P}$. Note the logarithmic scale of the two color axes. Values on the right side of each panel denote the RMS (mas) of the respective excitation function for $T<$ 120 days

### MITgcm LLC90 and LLC540 experiments

Two unconstrained MITgcm simulations were performed by us to assess the potential role of higher horizontal resolution for improving rapid OAM estimates. The first of these experiments, labeled LLC90, is based on the discrete ECCOv4 setup but uses first-guess (i.e., unadjusted) surface boundary conditions from ERA-Interim. The simulation was initialized from ECCOv4 climatologies for hydrographic variables and spun up for 6 months from a state of rest at 1 July 2006. Fifty layers of various thickness are used in the vertical, along with a nonlinear free surface and a time step of 30 min. Code and model parameters of the run slightly deviate from the ECCOv4 choices and are aligned with a hierarchy of LLC configurations derived from a global $^\circ$ parent grid and bathymetry ( Forget et al 2015, https://github.com/MITgcm-contrib/llc_hires, accessed 6 October 2020).

The setup for our eddy-permitting experiment, termed LLC540, is also part of the LLC suite and has a nominal horizontal resolution of $^\circ$. As in the LLC90 simulation, we use surface fluxes from ERA-Interim and allow for 6 months of spin-up, but set the time step to 10 min. Model energy is controlled by Leith biharmonic viscosity factors (non-dimensional values of 1.5)—see Fox-Kemper and Menemenlis (2008) for details—and a quadratic bottom friction coefficient of 0.0021 (also non-dimensional). Initial conditions for temperature and salinity were kindly provided by Hong Zhang (JPL) and descend from an ECCO state estimate on the LLC270 grid (Zhang et al 2018). In both experiments, we take the motion term from the model’s inline OAM integrator and estimate mass terms offline from 6-hourly instantaneous $p_b'$ fields (Eq. 6).

### DEBOT

We also consider results from a simple shallow-water model, referred to as DEBOT (David Einšpigel’s Barotropic Ocean Tide model, Einšpigel and Martinec 2017) and operated on a $^\circ$ latitude–longitude grid. The specific setup is that described by Schindelegger et al (2021) in the context of a study of global bottom pressure variability on sub-monthly time scales. ERA-Interim sea-level pressure and prognostic wind stress fields are used as forcing variables and dissipation is centered over topographic features through a linear wave drag scheme. From the two model runs conducted in Schindelegger et al (2021), we take the 2007–2009 integration without ocean self-attraction and loading (SAL) effects and extend it by 2 more years. Previous studies (e.g., Quinn et al 2019) have conjectured that the dynamic feedbacks associated with the SAL term might entail relevant OAM signals. However, based on a comparison of SAL vs. no-SAL simulation results over 2007–2009, we find that the effect is relatively weak in terms of broadband excitation values ($\sim$3 mas in ${\hat{\chi }}$, $\sim$3 $\upmu$s in $\chi _z$) and approximately a factor of 4 smaller than the residual polar motion and ${\Delta \Lambda }$ amplitude spectra after subtraction of atmospheric and oceanic signals.

### Other data sets

The Earth rotation data used in this study are the SPACE2018 series produced by Ratcliff and Gross (2019) from a combination of various space-geodetic Earth orientation measurements (Satellite and Lunar Laser Ranging, Very Long Baseline Interferometry, Global Positioning System). The solution compares favorably with geophysical excitation estimates across a broad range of frequencies and features no suspicious variability at sub-weekly periods (Dill et al 2020). We extract daily polar motion and ${\Delta \Lambda }$ values from the SPACE2018 version sampled at noon. Tidal contributions to length-of-day observations at 80 spectral lines are removed using the model by Ray and Erofeeva (2014). In want of a similarly refined treatment for long-period tidal effects in polar motion, we adopt estimates from Table 8.4 in Petit and Luzum (2010) but replace the dominant fortnightly component by the Mf solution of Ray and Egbert (2012).

A supplemental analysis of Southern Ocean bottom pressure variability in Sect. 4.3 draws on regularized daily GRACE gravity field determinations by ITSG (Institute of Geodesy at Graz University of Technology, release ITSG-Grace2018, Mayer-Gürr et al 2018; Kvas et al 2019). While being no “ground truth” in the strict sense of the word, these solutions provide a more realistic depiction of large-scale sub-monthly mass-field variability than their de-aliasing priors, down to periods of 4–5 days; see Schindelegger et al (2021) and references therein. As in the latter study, we map the ITSG-Grace2018 spherical harmonics coefficients for degrees $n = 2\ldots 40$ to gridded mass anomalies and add back the ocean background model (GAB product from MPIOM, including degree $n=1$, Dobslaw et al 2017). Daily solutions from 2007 to 2009 are considered, a period of homogeneous and near-continuous *K*-band range rate observations.

## Results

### OAM signal content

Before turning to excitation budgets, we highlight some characteristics of the modeled OAM functions, displayed as amplitude spectra of mass and motion components in Fig. 2. Axial terms allow for little discrimination among the five models and are omitted. Different estimates of $\chi ^m_{x,y}$ show a high degree of consistency and point to greater levels of excitation in the *y* component, rather than *x*, for periods longer than 10 days (cf. Gross et al 2003). A faint cusp of mass term variability at $\sim$5 days is likely the angular momentum signature of the ocean’s dynamic response to barometric pressure and wind stress fluctuations associated with the gravest symmetric mode of the Rossby–Haurwitz waves (Madden 2019), see also Ponte and Ali (2002). All models suggest pronounced bottom pressure effects in $\chi ^m_{y}$ around $T=20$ days, a peculiarity previously noted by Bizouard and Seoane (2010). We have performed complementary checks of $p_b'$ fields in that band (18–22 days) and found evidence for an out-of-phase relationship between the Indian and Pacific sectors of the Southern Ocean. Such geometry is generally conducive to a strong OAM signal in the *y* direction (cf. Sect. 4.3).

Examination of the LLC amplitude spectra and related RMS (root-mean-square) values at $T<$ 120 days, also included in Fig. 2, reveals that the LLC540 simulation yields weaker OAM variability than LLC90, especially at periods below 7 days. This is an interesting result, as the amount of damping conveyed by bottom friction and viscosity schemes (Sect. 3.3) is, to first order, identical between the two runs (D. Menemenlis, 2021, personal communication). Higher dissipation in LLC540 must stem from the sixfold increase in horizontal resolution, which can have various effects. First, grid spacing of $^\circ$ is sufficiently small to resolve the first baroclinic Rossby radius in the deep ocean within latitudes $|\phi |< 40^\circ$; cf. Figure 1 in Hallberg (2013). Transient eddy features are therefore admitted in the LLC540 simulation and tend to drain energy from the large-scale fields important to OAM quantities. Second, higher resolution necessarily results in sharper topographic gradients, which can enhance scattering of barotropic motions into baroclinic waves. In fact, localized interactions with topography have been shown to be relevant to the dynamic response of a stratified ocean to barometric pressure (Ponte and Vinogradov 2007), a process most active on sub-weekly time scale where LLC540 variability in $\chi ^{m,v}_{x,y}$ is weak.

A paramount feature in Fig. 2, particularly clear in the motion terms, are power deficits in the OAM functions from DEBOT. Assuming depth-independence of the flow field at large scales is generally justified on physical grounds (Willebrand et al 1980), but dynamics at a given latitude become more baroclinic with increasing period (Bingham and Hughes 2008). Hence, we expect a drop in coherence between DEBOT OAM estimates and rotation parameters toward the lower end of the frequencies considered. Most glaring though in Fig. 2 are large amplitudes in the MPIOM-based equatorial motion terms at periods of less than $\sim$2 weeks and 2–4 mas in excess relative to other models. Below, we offer more thoughts on these anomalies and their ramifications for modeling rapid polar motion.

### Excitation budgets

**Table 1:** Excitation budget for sub-seasonal Earth rotation changes over 2007–2011

	$\chi _x$	$\chi _y$	$\chi _x + \mathrm {i}\chi _y$	$\chi _z$
RMS of $\chi _j^G, j=\left( x,y,z\right)$	21.1	34.0	40.0	136.4
*PVE by atmosphere (IB) in* $\chi _j^G$				
ECMWF operational	50.1 (14.9)	55.9 (22.6)	54.2 (27.1)	93.7 (34.1)
ERA-Interim	48.8 (15.1)	54.3 (23.0)	52.8 (27.5)	93.6 (34.4)
*PVE by ocean in* $\chi _j^G - \chi _j^A$				
MPIOM	−15.6 (16.1)	13.3 (21.0)	4.5 (26.4)	60.2 (21.5)
ECCOv4	42.5 (11.5)	58.7 (14.7)	53.8 (18.7)	59.9 (21.8)
LLC90	41.1 (11.6)	56.7 (15.1)	52.0 (19.1)	56.8 (22.6)
LLC540$^\mathrm{a}$	50.7 (10.6)	72.3 (12.1)	65.7 (16.1)	65.0 (20.3)
$\hbox {DEBOT}^\mathrm{a}$	47.2 (11.0)	57.3 (15.0)	54.3 (18.6)	57.5 (22.4)
*PVE by sum of atmosphere (IB) and ocean in* $\chi _j^G$				
MPIOM & ECMWF operat.	42.3	61.7	56.3	97.5
ECCOv4 & ERA-Interim	70.6	81.1	78.2	97.5
LLC540 & ERA-Interim	74.8	87.3	83.8	97.8
*PVE by secondary terms in residual series* $\chi _j^G - \chi _j^A - \chi _j^O$				
HAM & SLAM (ESMGFZ)$^\mathrm{b}$	0.6	−0.1	0.2	4.2
Freshwater (ECCOv4)$^ \mathrm{b}$	−0.0	−0.1	−0.1	−2.5

Equatorial terms $\chi _x$ and $\chi _y$ (in mas) present the polar motion excitation budget, while the axial term $\chi _z$ (in $\upmu$s) is for ${\Delta \Lambda }$. Periods below 120 days are considered

Except for the first line (RMS of geodetic excitation), values are PVE, as specified by intermediate headers, and the corresponding RMS of residuals is added in parentheses where appropriate

$^\mathrm{a}$PVE by ocean in ${\hat{\chi }}^G$ (i.e., without subtracting IB-corrected atmospheric effects) is 36.1% for LLC540 and 33.0% for DEBOT

$^\mathrm{b}$Residual observed excitation computed either using MPIOM & ECMWF operational (for HAM and SLAM) or ECCOv4 & ERA-Interim (freshwater term)

**Fig. 3:**
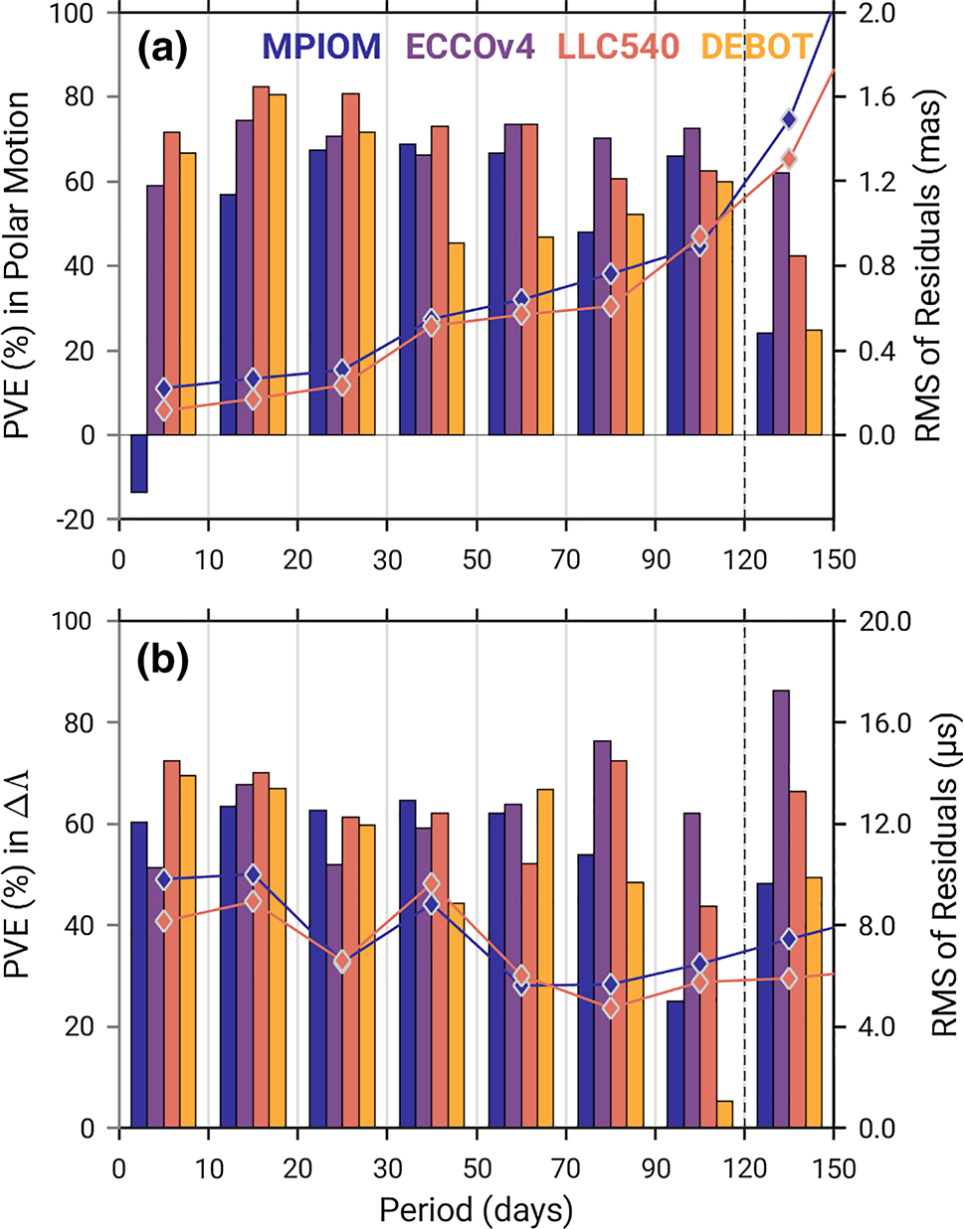
Evaluation of non-tidal oceanic contributions to **a** polar motion (mas) and **b** ${\Delta \Lambda }$ ($\upmu$s) in different spectral bands. Vertical bars, referred to by the left axes, show the PVE by four OAM series in atmosphere-corrected ${\hat{p}}$ and ${\Delta \Lambda }$, cf. also Table 1. LLC90 has been left out for visibility purposes but performs similarly to ECCOv4 for $T<$ 70 days. Solid lines display the RMS of the residual rotation series, after subtraction of atmospheric and oceanic (either MPIOM or LLC540) signals

Table 1 presents the paper’s main results in terms of the percentage of sub-seasonal excitation variance accounted for by geophysical excitation processes. For the ocean, these broadband statistics can be taken in together with a decomposition into bottom pressure and currents effects in Table 2 and a plot of PVE by four ocean models in ${\hat{p}}$ and ${\Delta \Lambda }$ changes over period, shown in Fig. 3. Note that the residual polar motion curves in Fig. 3a tend to follow the transfer function’s ascending slope ($\sigma \rightarrow 2\pi T_c^{-1}$, Eq. 2) and should be interpreted cautiously in quantitative terms. The baseline for assessing model “skill” is defined by residual geodetic excitation (or residual polar motion) with atmospheric contributions removed, i.e., $\chi _j^G - \chi _j^A$. Over 2007–2011, atmospheric processes—mostly tropospheric winds (Gross et al 2004)—explain as much as 93.7% of the observed non-tidal ${\Delta \Lambda }$ variance. They are less effective in exciting sub-seasonal wobbles (PVE of 54.2% in ${\hat{\chi }}$), consistent with Table 7 in Gross et al (2003).

Our assessment of oceanic effects contains several findings of value. First, geodesy’s leading OAM series given by MPIOM do not adequately reduce variance of the observed sub-seasonal polar motion excitation. Table 2 and Fig. 3a suggest that the deficiency has its source in the motion terms and at high frequencies, in line with the spectral characteristics of $\chi _{x,y}^v$ in Fig. 2. These short period fluctuations are absent from $\chi _z^v$ and inherently emphasized in evaluations of polar motion excitation based on deconvolution (Eq. 1, Chao 1985).

As a consistency check (not shown), we have computed proxy equatorial OAM motion terms from daily-averaged MPIOM bottom pressure fields (Dobslaw et al 2017). The analysis is based on time integration of relevant torques on the ocean in the frequency domain, similar to what we have previously tested for the atmosphere; cf. Schindelegger et al (2013). Benchmarks of the method with DEBOT along with results in Fujita et al (2002) indicate that a combination of ellipticity and seafloor topographic torques—both estimated from $p_b'$—account for most of the variance (PVE $=86$%) in the oceanic motion term. This first-order budget constraint is, however, poorly fulfilled in MPIOM (PVE $=44$%), implying the presence of a non-standard angular momentum source. Direct analysis of the model’s depth-integrated velocities could shed light upon the issue, as would an examination of equatorial friction torques. The latter are generally thought to be small (Fujita et al 2002) but might be inflated in MPIOM depending on the exact nature of dissipative closures and the offline bulk parameterization for air–sea momentum flux (Dobslaw and Dill 2019). While the origin of the high-frequency energy excess in MPIOM remains arcane, it is clear that these OAM series are not a good choice for evaluating the quality of geodetic polar motion determinations at sub-weekly periods (Dill et al 2020).

**Table 2:** PVE by oceanic excitation in atmosphere-corrected geodetic excitation ($T<$ 120 days, 2007–2011), split up into mass and motion terms for each $\hbox {model}$

	$\chi _x$	$\chi _y$	$\chi _x + \mathrm {i}\chi _y$	$\chi _z$
MPIOM				
Bottom pressure	18.2 (13.5)	54.7 (15.2)	43.5 (20.3)	48.1 (24.6)
Currents	−69.1 (19.4)	0.6 (22.5)	−20.6 (29.7)	32.0 (28.1)
ECCOv4				
Bottom pressure	22.6 (13.3)	49.7 (16.3)	41.5 (21.0)	45.0 (25.5)
Currents	1.2 (15.0)	34.1 (18.6)	24.1 (23.9)	32.4 (28.3)
LLC90				
Bottom pressure	25.7 (13.0)	52.2 (15.9)	44.2 (20.5)	45.7 (25.3)
Currents	−8.7 (15.8)	35.0 (18.5)	21.8 (24.3)	32.2 (28.3)
LLC540				
Bottom pressure	29.8 (12.7)	57.6 (15.0)	49.2 (19.6)	44.7 (25.6)
Currents	6.0 (14.7)	40.7 (17.7)	30.2 (23.0)	33.7 (28.0)
DEBOT				
Bottom pressure	21.2 (13.4)	47.7 (16.6)	39.7 (21.4)	40.7 (26.5)
Currents	9.6 (14.4)	32.1 (18.9)	25.3 (23.8)	28.1 (29.2)

Units and meaning of excitation quantities as in Table 1

Values are PVE by $\chi _j^O$ in $\chi _j^G - \chi _j^A$, where $j=\left( x,y,z\right)$, and RMS of residuals $\chi _j^G - \chi _j^A - \chi _j^O$, as shown in parentheses. Statistics for the combined effect of bottom pressure and currents are given in Table 1

None of the other statistics in Tables 1 and 2 suggests sizeable modeling issues. PVE calculated from ECCOv4, LLC90, and DEBOT fall in a narrow range (52.0–54.3% in ${\hat{\chi }}$, 56.8–59.9% in $\chi _z$), even though frequencies of closest correspondence to observations vary from model to model (Fig. 3). In particular, limitations of DEBOT’s constant-density formulation are evident outside the sub-monthly band, and data-constrained optimization in ECCOv4 provides for a better agreement with atmosphere-corrected rotation signals the greater the period. Improvements to OAM quantities with adjusted atmospheric forcing are most obvious in ${\Delta \Lambda }$, including a band (70–120 days) where the ${\Delta \Lambda } - \chi _z^A$ residual has been shown to be coherent with west equatorial Pacific wind stress variability (see Figure 3 in Marcus et al 2001). The potential role of optimization at these time scales could be clarified by dedicated analyses of changes in momentum flux, wind stress torque, bottom pressure, and depth-averaged horizontal currents relative to LLC90 or, better, an unconstrained ECCOv4 integration (cf. Ponte et al 2001).

In most, if not all of our broadband comparisons (Table 1), high PVE with LLC540 stand out. A residual RMS of 20.3 $\upmu$s in ${\Delta \Lambda }$ (both $\chi _z^A$ and $\chi _z^O$ removed) is marginally better (*p*-value $\sim 0.15$) than what MPIOM can give. More drastically, LLC540 accounts for 65.7% of the non-atmospheric polar motion excitation variance, with particular progress in modeling evident at periods below 50 days (cf. PVE of polar motion in Fig. 3a). DEBOT performs similarly for bands < 20 days, a result pointing to the benefits of higher horizontal resolution or enhanced dissipation, or both. As suggested in earlier work (Nastula and Ponte 1999; Ponte and Ali 2002), weak OAM variability, as long as with the right phases, is most commensurate with atmosphere-corrected polar motion excitation at rapid time scales. For ease of comparison with a similar analysis in Zhou et al (2005), we have recomputed similarity measures for the 4–20-day band. In this case, PVE by LLC540 (DEBOT) in ${\hat{\chi }}^G - {\hat{\chi }}^A$ is 72.7% (68.8%), compared to 51.6% from a barotropic ocean model in Zhou et al (2005).

Secondary excitation processes warrant a brief note. Most of the variability in HAM is compensated by barystatic effects (SLAM), so we evaluate their sum against $\chi _j^G - \chi _j^A - \chi _j^O$ using the ESMGFZ series. Additional OAM contributions in ECCOv4 due to net freshwater flux are readily computed as difference between the “yesFWF” and “noFWF” OAM functions available at the SBO website. Variance ratios of these terms relative to $\chi _j^O$ are around 1% for HAM/SLAM and $\ll$ 0.1% (1.6% in $\chi _z$) for ECCOv4 freshwater loads. Partly because of their smallness, neither component can account for appreciable variance in the residual geodetic excitation, see values in Table 1. Last, we note that only minor quantitative, but not qualitative, changes to our conclusions in this section were observed when another Earth rotation solution (IERS 14C04, Bizouard et al 2019)—or a different excitation formalism (Chen et al 2013)—was adopted for the comparison.

**Fig. 4:**
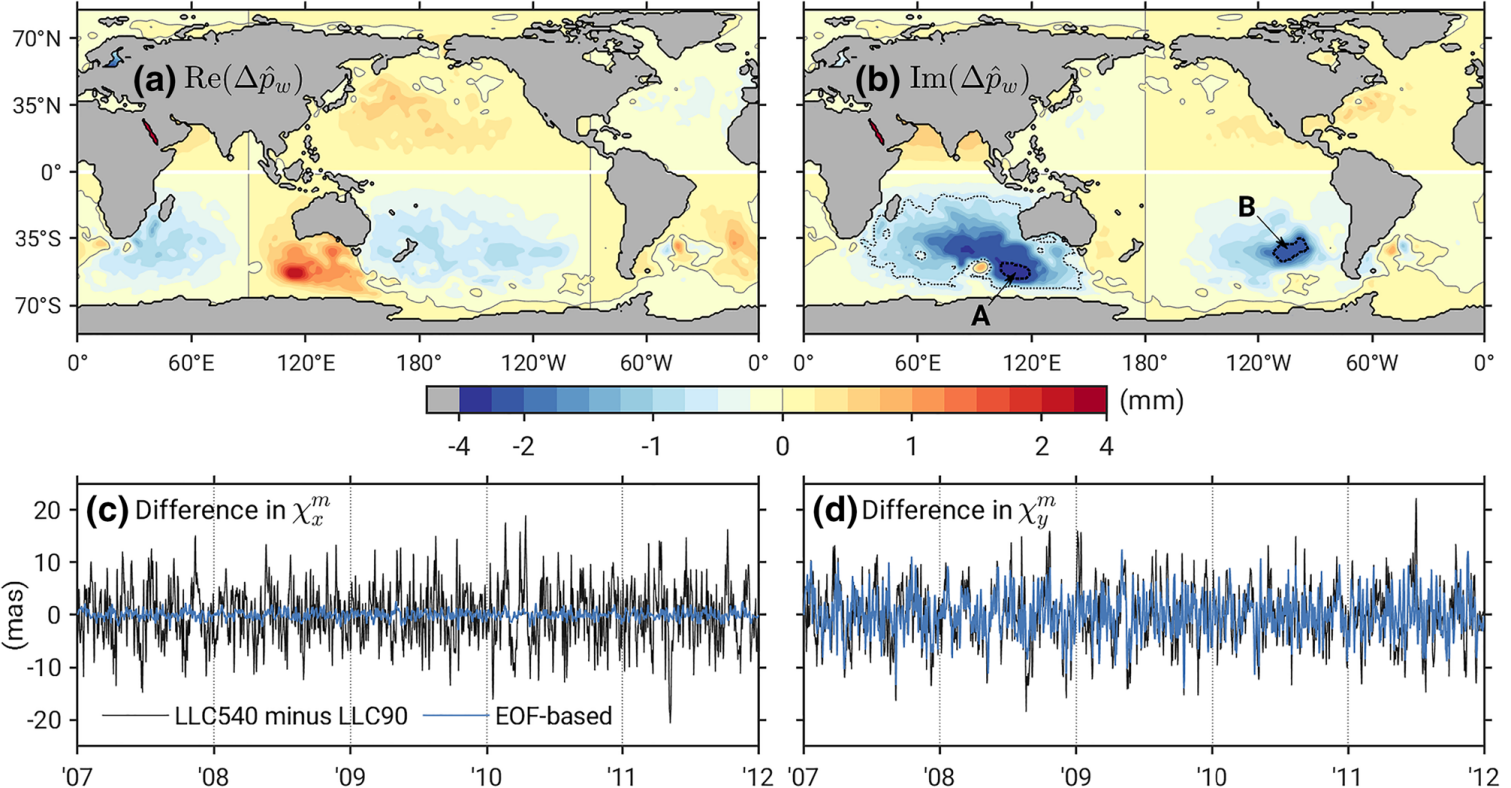
Connection of differences in the equatorial LLC mass terms (${\Delta }{\hat{\chi }}^m$, mas) to bottom pressure variability (mm of water height). **a**, **b** real and imaginary parts of that particular EOF mode in spatially weighted bottom pressure differences ${\Delta }{\hat{p}}_w$ (${\Delta }$ in the sense $\mathrm {LLC540}-\mathrm {LLC90}$) that generates the largest OAM signal. Blue lines in **c** and **d** show the corresponding signature of this mode in ${\Delta }{\hat{\chi }}^m$, which can be compared with the full difference (black lines) of the original LLC mass terms, analyzed in Sect. 4.2. See the main text for the significance of regions **A** and **B**. All illustrations are based on high-pass filtered bottom pressure and excitation time series ($T<$ 120 days)

### Insights from bottom pressure

Results above for the LLC simulations can be given additional context by mapping their differences in terms of $\chi$ functions in space and identifying areas that give the largest contribution to the global signal. Such analysis typically draws on gridded $\chi$ values for mass and velocity before their being summed up (e.g., Salstein and Rosen 1989; Nastula et al 2003, 2012). Here, we proceed along similar lines but (i) restrict ourselves to mass effects in the equatorial component, and (ii) work in units of bottom pressure instead of excitation functions. Specifically, we take differences of LLC540 relative to LLC90 in dynamic bottom pressure (${\Delta }p'_b$) and deduce complex-valued, spatially weighted bottom pressure differences

7 $$\begin{aligned} {\Delta }{\hat{p}}_w\left( \phi ,\lambda \right) = {\Delta }p'_b\left( \phi ,\lambda \right) \sin \phi \cos ^2\phi \mathrm {e}^{\mathrm {i}\lambda } \end{aligned}$$

by applying trigonometric weighting functions implicit to the products of inertia ${\hat{c}} = c_{xz} + \mathrm {i} c_{yz}$, multiplied with $\cos \phi$ from the grid point’s area element (cf. Nastula et al 2012). Prior to computing ${\Delta }p'_b$, different grid resolutions of the dynamic LLC fields were conformed using harmonic expansion to degree 179 and projection of the coefficients to a 1$^\circ$ target grid. With Eq. (7), the differential equatorial mass term (LLC540 minus LLC90) reads

8 $$\begin{aligned} {\Delta }{\hat{\chi }}^m = -a^4 \rho {\Omega } \sum _{\phi , \lambda } {\Delta }{\hat{p}}_w\left( \phi ,\lambda \right) \mathrm {d}\phi \mathrm {d}\lambda \end{aligned}$$

where *a* is Earth’s mean radius and $\rho$ is a reference density for seawater.

A generalized form of principal component (PC) analysis for vector quantities (Hardy and Walton 1978; Nastula et al 2003) was performed on filtered ${\Delta }{\hat{p}}_w$ ($T<$ 120 days) to separate these anomalies into complex-valued spatial modes (Empirical Orthogonal Functions, EOFs) and their time-dependent modifications (PCs). We rank the so derived modes by their variance in globally integrated excitation functions (Eq. 8), upon a synthesis step involving both the EOFs and the corresponding PCs. Summing up *all* modes according to Eq. (8) gives the original ${\Delta }{\hat{\chi }}^m$ time series, testifying to the correctness of our method.

Panels a and b in Fig. 4 display the spatial pattern of the leading mode in the re-ranked EOF spectrum, which accounts for 26% (2% in *x*, 49% in *y*) of the variance in ${\hat{\chi }}^m$ differences between LLC540 and LLC90 (panels c and d). The mode’s energy is concentrated in the imaginary part, resulting in a relatively large standard deviation of 3.9 mas in $\chi _y^m$ since the associated PC has a negligible imaginary component. That this differential excitation signal is important for improving the agreement with rotation data is highlighted in Table 3, where we re-evaluate the LLC90 polar motion excitation budget with the model’s original mass terms corrected such that they include the contributions shown as blue lines in Fig. 4c, d. The correction reduces the RMS of $\chi _y$ residuals from 15.1 mas to 13.9 mas; cf. 12.1 mas with LLC540 in Table 1. Differences in the *x* OAM functions contain contributions from several other EOFs and are not discussed here.

**Table 3:** Extension of Table 1 for LLC90 with modified mass $\hbox {terms}$

	$\chi _x$	$\chi _y$	$\chi _x + \mathrm {i}\chi _y$
*PVE by ocean in* $\chi _j^G - \chi _j^A$ *(RMS of residuals)*			
LLC90, original	41.1 (11.6)	56.7 (15.1)	52.0 (19.1)
LLC90, variant 1$^\mathrm{a}$	41.7 (11.5)	63.4 (13.9)	56.8 (18.1)
LLC90, variant 2$^\mathrm{b}$	41.0 (11.6)	61.9 (14.2)	55.6 (18.3)

Evaluation metrics and excitation quantities (in units of mas) as in Table 1

$^\mathrm{a}$Superimposed on the original LLC90 mass terms is the excitation signal associated with the main EOF mode in spatially weighted bottom pressure differences ${\Delta }{\hat{p}}_w$ (LLC540 minus LLC90). Figure 4 illustrates this statistical mode and its contribution to $\chi _x^m$ and $\chi _y^m$

$^\mathrm{b}$Same as variant 1, but with the spatial integration of the mode toward OAM values restricted to the South Indian Ocean, delimited by the dotted black contour in Fig. 4b

Can we make physical assertions based on the spatial pattern depicted in Fig. 4b? To some extent. The EOF’s negative maxima in the Southern Ocean coincide with the centers of the Australian-Antarctic Basin (**A**) and the Chile Rise (**B**) in the Bellingshausen Basin, i.e., areas known for their high levels of barotropic variability on intraseasonal time scales (Fukumori et al 1998; Fu 2003). These deep basins are encircled by closed, or almost closed, contours of potential vorticity , where *f* is the Coriolis parameter and *H* the local water depth. The specific  distribution facilitates a near-resonant response to wind stress curl with characteristic decay time scales of about 4 days (Weijer 2010, 2015). At constricted topographic features, energy of the mode is expended to a residual flow that may dissipate elsewhere, e.g., the greater South Indian Ocean surrounding area **A**. Figure 4b, paired with Fig. 6 in “Appendix,” indicates that the trapped modal circulation and its leakage to areas outside the Australian-Antarctic basin are of too large magnitude in LLC90. The most consistent explanation of this picture is that the model’s 1$^\circ$ horizontal grid inhibits a proper representation of topographic effects that set the near-resonant response and its energetics.

**Fig. 5:**
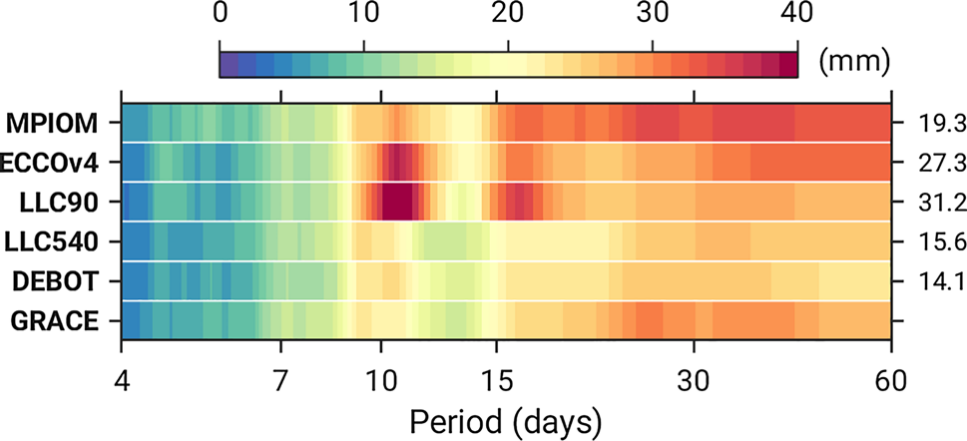
Amplitude spectra of area-averaged bottom pressure variations (mm) over the center of the Australian-Antarctic Basin (**A**) from models and daily GRACE solutions. Values on the right denote RMS differences (mm) between GRACE and the respective model at periods of 4–60 days. Spectra were computed with the same method as in Fig. 2 but over the time span 2007–2009

Figure 5 underpins the point in the frequency domain and across all models analyzed. We show amplitude spectra of area-averaged $p_b'$ series in region **A**, along with similar estimates from daily GRACE solutions at periods from 4 to 60 days. All bottom pressure series imply enhanced variability in the same bands, most notably near 10 days (cf. Fukumori et al 1998). DEBOT and LLC540 are tightly aligned with spectral characteristics in GRACE, a result also borne out by their low RMS differences with satellite-based $p_b'$ fluctuations (statistics are included in Fig. 5). In contrast, all 1$^\circ$ models—especially LLC90—are subject to a systematic excess in power from 10 to 16 days, consistent with some of the main periodicities seen in the LLC mass term differences ($\chi _y^m$, Fig. 4d). Although rather local in character, these model comparisons and insights from satellite gravimetry provide, at the very least, valuable hints on where to improve bathymetry in LLC90 (and thus ECCOv4) for better agreement with observed polar motion excitation. Evidently, a more complete analysis of ocean model differences and regional sources of excitation calls for scrutiny of volume transports and possible cancellation between mass and motion effects in the global OAM integrals.

## Summary remarks and outlook

We have taken a fresh look at the ocean’s involvement in the planetary angular momentum budget on time scales from days to months. Emphasis has been on judging numerical model skill and, to a lesser extent, on interpreting differences between various OAM estimates in terms of ocean dynamics. While evaluations of length-of-day changes spared major surprises, headway was made in accounting for Earth’s sub-seasonal wobbles. A key quantitative result is that joint consideration of AAM and OAM explains as much as 83.8% of the variance in equatorial geodetic excitation (Table 1). Atmospheric processes still dominate the relationship, but margins relative to oceanic contributions (PVE of 52.8% by ${\hat{\chi }}^A$ vs. 36.1% by ${\hat{\chi }}^O$, see Table 1) are evidently tighter than previously thought (Gross et al 2003).

Much of these advances have been facilitated by extending the scope of inquiry from current standard products (e.g., MPIOM, ECCO) to ocean models with higher horizontal resolution and more nuanced dissipation properties. In particular, excitation budgets in Sect. 4.2 present—to our knowledge—the first thorough assessment of angular momentum series from an eddy-permitting ocean model (LLC540) and a shallow-water model with drag concentrated over relief (DEBOT). Explorations of this kind are important, as they inform broader OAM modeling efforts (by ESMGFZ and ECCO) of avenues that are likely to give reward. Results here and elsewhere (Weijer 2010; Schindelegger et al 2021) suggest that refining horizontal grid spacing from 1$^\circ$ to $\sim$ $^\circ$ improves the representation of barotropic variability in the Southern Ocean, which projects strongly on polar motion. Transitioning of the ECCO state estimates to the LLC270 grid is underway (Zhang et al 2018) and will cast light on this very issue.

Further increases in spatial resolution will give rise to a more active mesoscale eddy field, which can substantially change bottom velocities and pathways of kinetic energy in general (Thoppil et al 2011). Experiments with wave drag on the near-bottom flow in eddying environments exist (e.g., Trossman et al 2013), and such model adaptations could also be relevant in a global OAM context. Nonetheless, the level of agreement with rotation data documented here with LLC540 implies that standard dissipation schemes—i.e., quadratic bottom friction and biharmonic horizontal viscosity—are a good starting point even when grid spacing is considerably refined.

Pure forward models aside, the merits of higher resolution may also be explored in novel eddy-resolving ($^\circ$) ocean reanalyses that span more than 25 years (Lellouche et al 2018). Whether or not such reanalyses can enhance the realism of OAM estimates in a manner we know from the atmosphere remains to be seen. A potential pitfall of sequential data assimilation schemes is unphysical changes in state variables when least-squares fits to sparsely sampled observations are carried out in predefined analysis intervals (Wunsch and Heimbach 2013). Property conservation and kinematic consistency of a geophysical fluid’s reconstructed state have high priority in Earth rotation research. On that account, the geodetic community will benefit from ongoing work with self-consistent smoother methods, as practiced by the ECCO consortium (Heimbach et al 2019; Quinn et al 2019).

## Figures and Tables

**Fig. 6:**
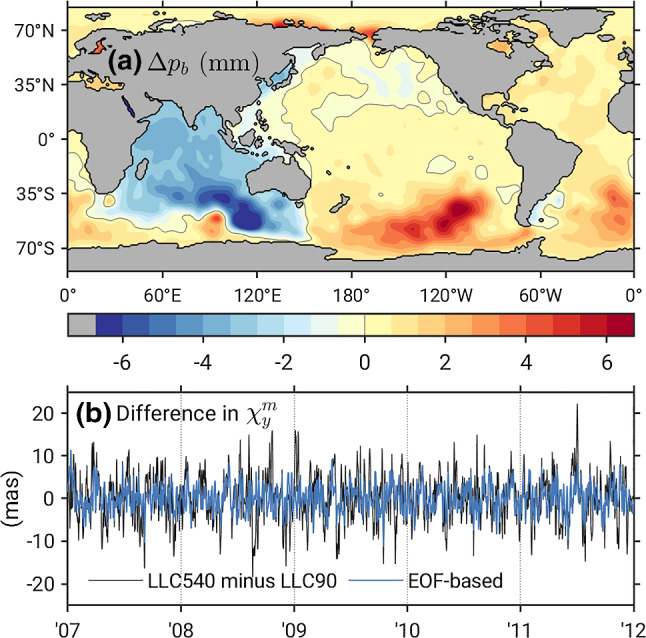
As Fig. 4 in the main text, but with the PC analysis performed on unweighted bottom pressure differences ${\Delta }{p}_b'$ (mm). **a** EOF mode that causes the largest contribution to the raw LLC mass term differences (${\Delta }{\hat{\chi }}^m$, mas). The mode explains 29.9% of the differential excitation signal in $\chi _y^m$ (blue vs. black lines in **b**)

## Data Availability

The LLC540 OAM series (2007–2011) are available from the IERS SBO website (https://isdc.gfz-potsdam.de/ggfc-oceans/). Daily bottom pressure anomalies from the two LLC simulations, synthesized on a 1$^\circ$ grid from spherical harmonics, have been deposited at https://doi.org/10.5281/zenodo.4707150. All other data sets used in this study are accessible through the following links: ESMGFZ angular momentum functions (http://rz-vm115.gfz-potsdam.de:8080/repository/), ITSG-Grace2018 bottom pressure (https://ifg.tugraz.at/ITSG-Grace2018), MPIOM dynamic bottom pressure (ftp://isdcftp.gfz-potsdam.de/grace/Level-1B/GFZ/AOD/RL06/), ECCOv4 dynamic bottom pressure (https://ecco-group.org/products-ECCO-V4r4.htm), code customization and scripts of the LLC family (https://github.com/MITgcm-contrib/llc_hires), ERA-Interim (https://apps.ecmwf.int/datasets/data/interim-full-daily/).

## A Appendix

An alternative way of identifying oceanic areas relevant to equatorial mass term changes is to perform the PC analysis on original (unweighted and real-valued) pressure data ${\Delta }{p}_b'$, rather than ${\Delta }{\hat{p}}_w$ as in Section 4.3. After re-ranking the (real-valued) EOF spectrum by each mode’s OAM contribution, we obtain a leading mode as shown in Fig. 6. The results reiterate the significance of topographically controlled variability in the Southern Ocean for explaining LLC mass term differences and provide an additional link between Fig. 4 (decomposition of ${\Delta }{\hat{p}}_w$) and the local $p_b'$ diagnostics of Fig. 5.
